# Boarding Schools as Disseminators of Disease

**Published:** 1895-03-02

**Authors:** 


### Editor's Letter-Box.

[Our correspondents are reminded that prolixity is a bar to publication, and that brevity of style and conciseness of statement greatly facilitate early insertion.]

### BOARDING SCHOOLS AS DISSEMINATORS OF DISEASE.

"The Mother of Holiday House" writes: I must beg to thank you for your excellent article in this week s Hospital on "Boarding Schools as Disseminators of Diseases." I trust the attention of the public and of head masters and mistresses will be called to the important point raised in it, *i.e.*, that children should bring a health certificate home as well as take one to school with them. For the last nineteen years I have had to do with holiday children, and have often remarked the careless, casual way schoolmasters send them home from a school where measles, chicken-pox, and the like have been prevalent during the term. Sometimes with no official notification of the fact, but generally a friendly note is sent the day before the child arrives, saying, "We have had a few cases of such-and-such; take what precautions you like as to disinfection or quarantine." This particularly friendly form was used by a house master in one of our large public schools, leaving the poor parents less than twenty-four hours to find suitable quarantine quarters, and of course spreading, or renewing the chance of spreading, the infectious complaint throughout numberless houses, to say nothing of railway carriages, cabs, or fellow-travellers. A few summers ago I had eight children spending their holidays with me, some my own, some boarders, and some holiday pupils. Seven had to return to different schools. One school kindly sent us chicken-pox, with only a friendly note with the boy. Four of my eight developed it one after another, and it took nine weeks to get through with it, rid of it, and get the doctor to give a clean bill of health to the girls and boys who had been in the house! Besides spoiling the fun and happiness of the holidays for all the young folk, and having the house sent to Coventry by all the neighbours, this caused doctors' bills, and a great deal of worry and anxiety for the heads of the establishment. And all because schools do not practice what they preach, or, in other words, do as they are done by. At first sight it might appear unfair to expect schools to keep the pupils until they are free from infection, but the question is as broad as it is long, or nearly so. Parents have to pay a full term's fees whether the child returns the beginning of term or not; and, besides feeding him, probably pay for a tutor or governess to prevent him losing places at school by the enforced quarantine. Here the schoolmaster gains and the parents lose. When the infection is at school this could be reversed, or more likely the parents would willingly pay by the week till a clean bill of health could be given rather than have infection brought home to the rest of the family. So that, except the lengthened responsibility, the master would be the gainer under either condition! There is another point, sir, to which I should like to draw your attention, and that is the increasing custom of giving exeats. I have noticed time after time at the different schools I am acquainted with that after the half-term's exeat, very seldom before, some infectious complaint breaks out. Why do schools allow exeats? The terms are short, the holidays long! Very different to thirty years ago when school boys came home only at Christmas and midsummer, really midsummer it was then. Then an exeat might have been expected, but it was unheard of. Now, it seems, children are allowed exeats always once, sometimes twice, a term—whether convenient or the reverse to the parents—and the question of health certificates are never mentioned, although of such paramount importance to all! And how are exeats generally spent? In taking the boys and girls to entertainments, theatres, circus, and what not. So that four risks of infection are run for three or four days' outing: 1st, the journey home; 2nd, the home; 3rd, the entertainments; and 4th, the journey back to school. And no one asks for a health certificate. Surely the certificate at the commencement of the term is a mere farce if the exeat follows in four or five weeks. Let all parents and school masters set their faces against this modern and unnecessary custom, if infectious diseases are to be stamped out from among us. Girls' school sin more than boys in this particular, I notice. Of course, we are delighted to see our boys and girls home, although we disapprove of the system of exeats. Still, surely the parents could just as easily and far more wisely visit the children at their school if they cannot endure separation for ten to twelve weeks at most. And I am not at all sure whether such a practice would not be very advantageous to some schoolmasters and mistresees. I again thank you for having ventilated the subject, and hope that it will be thoroughly taken up by parents generally throughout the country, not only for our own sakes, but also with a view to prevent as much as possible the spread of infectious diseases.

